# Hypothalamic lipoma and growth hormone deficiency

**DOI:** 10.1186/s13633-020-0074-9

**Published:** 2020-02-05

**Authors:** Anne Rochtus, Joseph Vinckx, Francis de Zegher

**Affiliations:** 1 Pediatric Neurology, University Hospitals Leuven, 3000 Leuven, Belgium; 2 Pediatric Endocrinology, University Hospitals Leuven, Herestraat 49, 3000 Leuven, Belgium

**Keywords:** Hypothalamic lipoma, Growth hormone deficiency, Intracranial lipoma, Child

## Abstract

**Background:**

Intracranial lipomas are rare, congenital lesions, most often located at the midline. Most hypothalamic lipomas are asymptomatic, but some cases have been associated with precocious puberty, hypothermia, headache and/or obesity.

**Case presentation:**

A 7-year-old boy was referred for short stature and proved to be partially growth-hormone deficient. Magnetic resonance imaging (MRI) revealed a lipoma in the paramedian hypothalamus. Growth hormone treatment resulted in swift and uncomplicated catch-up growth.

**Conclusions:**

The present case appears to be the first to link hypothalamic lipoma to GH deficiency. The neuro-endocrine pathophysiology underpinning this link remains to be explored.

## Established facts and novel insights

**Table:** 

Established Facts	
• Intracranial lipomas are generally benign, congenital malformations, most often located at the midline of the central nervous system.	
• Hypothalamic lipomas are most often asymptomatic, but can be associated with precocious puberty, hypothermia, headache and/or obesity.	
Novel Insight	
Hypothalamic lipoma can also present with growth failure due to a growth hormone (GH) deficiency that responds well to treatment with rhGH.	

## Introduction

Intracranial lipomas are rare congenital lesions, accounting for less than 1% of all intracranial lesions [1–4]. They are thought to result from an abnormal persistence of the meninx primitiva, the mesenchymal tissue that gives rise to the meninges, and its subsequent differentiation into adipose tissue [4]. Most intracranial lipomas are located at the midline, in the interhemispheric fissure (45%), or in the quadrigeminal/superior cerebellar cistern (25%). The remainder of the lesions are found in the cerebellopontine angle cistern (9%) and sylvian cistern (5%) [2]. More than half of the intracranial lipomas are associated with brain malformations, often midline anomalies. Most often they are asymptomatic and found incidentally during autopsy or neuroradiologic investigations for other conditions. They are usually stable or slowly growing, but long-term follow-up is lacking [5]. Symptoms differ by the location of the lipoma. Here, we present a first case linking a hypothalamic lipoma to growth hormone deficiency.

## Case report

**Fig. 1:**
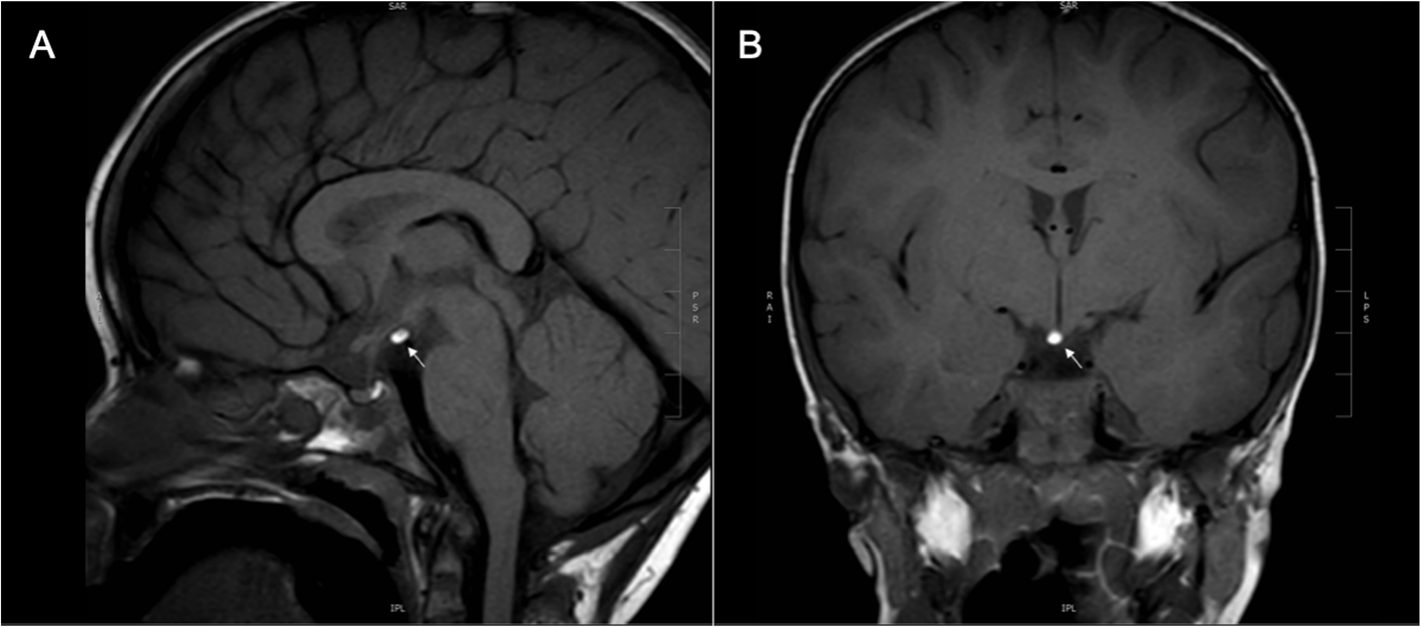
Sagittal (**a**) and coronal (**b**) T1-weighted MR images reveal a hypothalamic lipoma. The pituitary gland has a normal signal intensity and no other intra-cranial abnormalities are found

**Fig. 2:**
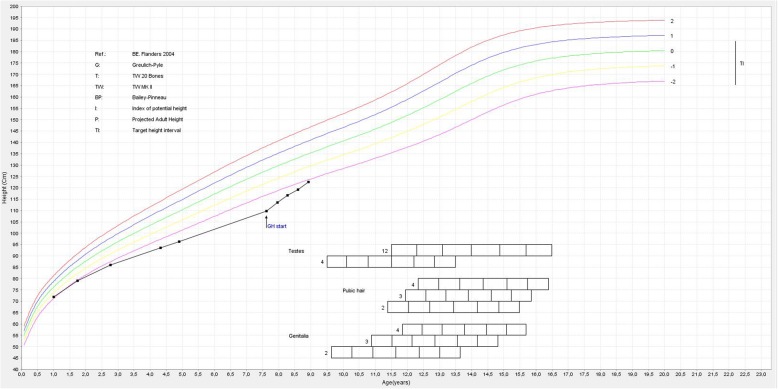
Graph showing the progression of the patient’s height up to age 8.9 years. The values on the x axis represent age in years, those on the y axis represent height in centimeters. GH start: start of growth hormone therapy; TI: target height interval

A 7.6-year-old boy was referred for investigation of short stature. He was born after 39 weeks of gestation, with a birth weight of 3.38 kg (0.0 SD) and birth length of 47 cm (− 1.8 SD). Since the age of 1 year, height SD had gradually decreased from − 1.7 to − 3.3 SD, whereas the mid-parental height was − 0.8 SD. He did not have polydipsia or polyuria. The boy had previously been diagnosed with an autistic spectrum disorder, but his clinical examination was otherwise unremarkable; he had normal genitalia and there were no signs of puberty (Tanner stages A1 P1 G1, testes 1/1 ml). There was no familial history of constitutional delay. Endocrine results were suggestive of partial GH deficiency (circulating insulin-like growth factor-I 48 μg/L (− 2.5 SD) [6]; peak GH of 8.9 μg/L after glucagon). TSH was borderline elevated but free T4 was normal (TSH 5.3 mIU/L, free T4 14.6 pmol/L), and basal concentrations of circulating cortisol (8.8 μg/dL), dehydroepiandrosterone-sulphate (DHEAS 0.6785 μmol/L), luteinizing hormone (LH < 0.1 IU/L), follicle stimulating hormone (FSH 0.8 IU/L) and prolactin (4.7 μg/L) were unremarkable. MRI of the brain revealed a lipoma on the midline adjacent to the hypothalamus (Fig. 1). The appearance of the anterior and posterior pituitary gland was normal. GH replacement treatment significantly increased height velocity from 5.2 cm/year to 9.4 cm/year in the first year of catch-up growth (Fig. 2).

## Discussion

We present a first case associating hypothalamic lipoma with prepubertal partial GH deficiency. Hypothalamic lipoma used to be an incidental autopsy finding [7–9]. Only 5 symptomatic cases of hypothalamic lipoma have been reported, respectively with precocious puberty [10], hypothermia [11], headaches [12] and/or obesity [13]. It has long been established that children with a hypothalamic hamartoma, another congenital benign mass lesion in the ventral hypothalamus, may present with neuro-endocrine features such as central precocious puberty, gelastic seizures, cognitive deficit or psychiatric problems. It is hypothesized that the clinical phenotype of hypothalamic hamartoma relates directly to the region of the hypothalamus to which it anatomically connects: connection to the posterior hypothalamus (mammillary bodies and limbic circuit) may result in epilepsy, whereas connection to the anterior hypothalamus (pituitary stalk and tuber cinereum) may cause central precocious puberty, which is thought to be caused by ectopic generation and pulsatile release of gonadotropin-releasing hormone (GnRH) from the hamartoma [12]. Martin et al. [14] reported a single patient with a hypothalamic hamartoma and a combination of GH deficiency and hypogonadotropic hypogonadism. Lesions of the ventromedial nucleus are known to result in poor growth due to a reduction in GH release [15]. The neuro-endocrine mechanisms responsible for GH deficiency within a context of hypothalamic hamartoma or lipoma remain to be delineated.

## Conclusion

Hypothalamic lipoma is a rare congenital intracranial lesion. A first prepubertal child with a hypothalamic lipoma and partial GH deficiency has been identified.

## Data Availability

All data generated or analysed during this study are included in this published article.

## Abbreviations

DHEAS: Dehydroepiandrosterone-sulphate
FSH: Follicle stimulating hormone
GH: Growth hormone
GnRH: Gonadotropin-releasing hormone
IGF-I: Insulin-like growth factor
LH: Luteinizing hormone
MRI: Magnetic resonance imaging
SD: Standard deviation

## References

[1] Loddenkemper T, Morris HH, Diehl B, Lachhwani DK (2006). Intracranial lipomas and epilepsy. J Neurol.

[2] Jabot G, Stoquart-Elsankari S, Saliou G, Toussaint P, Deramond H, Lehmann P (2009). Intracranial lipomas: clinical appearances on neuroimaging and clinical significance. J Neurol.

[3] Yildiz H, Hakyemez B, Koroglu M, Yesildag A, Baykal B (2006). Intracranial lipomas: importance of localization. Neuroradiology.

[4] Truwit CL, Barkovich AJ (1990). Pathogenesis of intracranial lipoma: an MR study in 42 patients. Am J Neuroradiol.

[5] Joigneau Prieto L, Ruiz Y, Pérez R, De León Luis J (2019). Prenatal diagnosis of pericallosal lipoma: systematic review. Eur J Paediatr Neurol.

[6] Bidlingmaier M, Friedrich N, Emeny RT, Spranger J, Wolthers OD, Roswall J (2014). Reference intervals for insulin-like growth factor-1 (IGF-I) from birth to senescence: results from a multicenter study using a new automated chemiluminescence IGF-I immunoassay conforming to recent international recommendations. J Clin Endocrinol Metab.

[7] Moschopulos M, Becheanu G, Stamm B (2006). Hypothalamic osteolipoma of the tuber cinereum. J Cell Mol Med.

[8] Chu AY, Rorke LB, Hood IC (2005). Lipoma of the tuber cinereum. Arch Pathol Lab Med.

[9] Wittig H, Kasper U, Warich-Kirches M, Dietzmann K, Roessner A. Hypothalamic osteolipoma. A case report. Gen Diagn Pathol. 1997;142(5-6):361-4. 9228262

[10] Bognár L, Bálint K, Bárdóczy Z (2002). Symptomatic osteolipoma of the tuber cinereum: case report. J Neurosurg.

[11] Di Pietro P, Debbia C, Fondelli MP (2004). Pediatric hypothalamic lipoma with hypothermia - case report. Brain and Development.

[12] Kurt G, Dogulu F, Kaymaz M, Emmez H, Önk A, Baykaner MK (2002). Hypothalamic lipoma adjacent to mamillary bodies. Childs Nerv Syst.

[13] Puget S, Garnett MR, Leclercq D, Pinto-Primard G, Samara-Boustani D, Sainte-Rose C (2009). Hypothalamic lipoma associated with severe obesity: report of 2 cases. J Neurosurg Pediatr.

[14] Martin DD, Seeger U, Ranke MB, Grodd W (2003). MR imaging and spectroscopy of a tuber cinereum hamartoma in a patient with growth hormone deficiency and hypogonadotropic hypogonadism. Am J Neuroradiol.

[15] Murray PG, Higham CE, Clayton PE (2015). The hypothalamo-GH axis: the past 60 years. J Endocrinol.

